# Association of Human Antibodies to Arabinomannan With Enhanced Mycobacterial Opsonophagocytosis and Intracellular Growth Reduction

**DOI:** 10.1093/infdis/jiw141

**Published:** 2016-04-07

**Authors:** Tingting Chen, Caroline Blanc, Anke Z. Eder, Rafael Prados-Rosales, Ana Camila Oliveira Souza, Ryung S. Kim, Aharona Glatman-Freedman, Maju Joe, Yu Bai, Todd L. Lowary, Rachel Tanner, Michael J. Brennan, Helen A. Fletcher, Helen McShane, Arturo Casadevall, Jacqueline M. Achkar

**Affiliations:** 1 Department of Medicine; 2 Department of Microbiology and Immunology; 3 Department of Epidemiology and Population Health, Albert Einstein College of Medicine, Bronx; 4 Department of Pediatrics; 5 Department of Family and Community Medicine, New York Medical College, Valhalla, New York; 6 Aeras, Rockville; 7 Department of Molecular Microbiology and Immunology,Johns Hopkins Bloomberg School of Public Health; 8 Department of Medicine, Johns Hopkins University School of Medicine, Baltimore,Maryland; 9 Infectious Diseases Unit, Israel Center for Disease Control, Israel Ministry of Health, Tel Hashomer; 10 Alberta Glycomics Centre; 11 Department of Chemistry, University of Alberta, Edmonton, Canada; 12 Jenner Institute, University of Oxford, United Kingdom

**Keywords:** tuberculosis, *Mycobacterium tuberculosis*, *Mycobacterium bovis* BCG, immunoglobulin, human antibodies, antibody-mediated immunity, immune response, polysaccharide, oligosaccharide, bacterial capsule

## Abstract

***Background.*** The relevance of antibodies (Abs) in the defense against *Mycobacterium tuberculosis* infection remains uncertain. We investigated the role of Abs to the mycobacterial capsular polysaccharide arabinomannan (AM) and its oligosaccharide (OS) fragments in humans.

***Methods.*** Sera obtained from 29 healthy adults before and after primary or secondary bacillus Calmette-Guerin (BCG) vaccination were assessed for Ab responses to AM via enzyme-linked immunosorbent assays, and to AM OS epitopes via novel glycan microarrays. Effects of prevaccination and postvaccination sera on BCG phagocytosis and intracellular survival were assessed in human macrophages.

***Results.*** Immunoglobulin G (IgG) responses to AM increased significantly 4–8 weeks after vaccination (*P* < .01), and sera were able to opsonize BCG and *M. tuberculosis* grown in both the absence and the presence of detergent. Phagocytosis and intracellular growth inhibition were significantly enhanced when BCG was opsonized with postvaccination sera (*P* < .01), and these enhancements correlated significantly with IgG titers to AM (*P* < .05), particularly with reactivity to 3 AM OS epitopes (*P* < .05). Furthermore, increased phagolysosomal fusion was observed with postvaccination sera.

***Conclusions.*** Our results provide further evidence for a role of Ab-mediated immunity to tuberculosis and suggest that IgG to AM, especially to some of its OS epitopes, could contribute to the defense against mycobacterial infection in humans.

Active tuberculosis remains a major public health problem, with around 9 million new cases and 1.5 million associated deaths globally [1]. The only licensed vaccine, bacillus Calmette-Guerin (BCG), confers limited protection against pulmonary tuberculosis, and does not prevent *Mycobacterium tuberculosis* infection [2]. Tuberculosis vaccines under development aim to enhance cell-mediated immunity but, as of today, have not demonstrated sufficient protective efficacy in humans [2]. Thus, further exploration of other arms of the immune system may inform new vaccine development strategies.

Increasing evidence suggests that antibodies (Abs) could contribute to the defense against *M. tuberculosis* [3, 4]. However, their role is insufficiently studied because of the general perception that *M. tuberculosis*, a largely intracellular pathogen, is outside the reach of extracellularly located Abs. Abs against capsular and noncapsular surface polysaccharides are protective against a variety of microbial pathogens, including those with intracellular location [4, 5]. Mycobacteria have a capsule, an important virulence factor, that consists largely of polysaccharides, proteins, and, to a smaller extent, glycolipids [6, 7]. α-glucan and arabinomannan (AM) account for 70%–80% and 10%–20% of the polysaccharide content, respectively [8, 9]. We, as well as others, have previously shown that AM is more immunogenic than α-glucan in patients with tuberculosis [10, 11]. We have further demonstrated that Ab responses to capsular AM and the related cell wall glycolipid lipoarabinomannan (LAM) correlate strongly and significantly in patients with tuberculosis [10], suggesting, in accordance with other studies [12], that AM is the immunogenic portion of LAM. Of note, LAM is not a component of the mycobacterial capsule [8, 9]. Importantly, we and others have shown that passive administration of monoclonal Abs (mAbs) to AM and the AM portion of LAM improve the outcome of *M. tuberculosis* infection in mice [12, 13] and that AM-containing conjugate vaccines result in high immunoglobulin G (IgG) titers to AM and protection of mice against *M. tuberculosis* infection (unpublished data) [14, 15]. In humans, an increase in LAM-specific Abs after BCG vaccination has been associated with enhanced innate and cell-mediated immune responses against BCG [16]. Furthermore, a lack of Abs to LAM was associated with tuberculosis dissemination in children [17]. These experimental and clinical data support the notion that Abs to AM might play a protective role against *M. tuberculosis* infection in both animal models and humans.

In this study, we tested Ab responses to capsular AM in sera obtained before and after BCG vaccination from adult subjects undergoing a clinical study to assess mycobacterial growth inhibition assays (MGIAs) as correlates for vaccine efficacy [18]. We hypothesized that BCG vaccination would lead to increased Ab titers to capsular AM, particularly to some of its oligosaccharide (OS) epitopes, and that such Ab responses would be associated with defensive effects against mycobacterial infection.

## MATERIALS AND METHODS

### Subjects

Sera were obtained from 30 healthy, *M. tuberculosis*–uninfected adults before and after (weeks 4, 8, and 24) primary or secondary BCG vaccination at the University of Oxford (Oxford, United Kingdom) [18]. Participants were recruited under a protocol approved by the Oxfordshire Research Ethics Committee [18], and peripheral blood mononuclear cells (PBMCs) from independent healthy BCG vaccinated subjects were collected under a protocol approved by the Institutional Review Board of the Albert Einstein College of Medicine. Written informed consent was obtained from all individuals prior to enrollment.

### Enzyme-Linked Immunosorbent Assays (ELISAs)

Capsular AM from BCG (Pasteur) or *M. tuberculosis* (H37Rv) was isolated, purified, and lyophilized as described [19]. LAM, isolated from the cell wall of *M. tuberculosis* H37Rv, was obtained from BEI Resources (NR-14848). Maxisorp plates were coated with AM or LAM at 10 μg/mL, sera were added at 1:50 dilution, and ELISAs were performed as described elsewhere [10], with further details in the Supplementary Methods.

### AM Microarrays

A panel of 12 AM fragments (corresponding to motifs at the nonreducing terminus of the molecule, which have previously been shown to be recognized by anti-AM/LAM Abs) [20–22] were synthesized, coupled to bovine serum albumin via a squarate linker [23], and printed on epoxy-coated glass slides [24]. After blocking, the slides were incubated with sera (1:100), the murine mAb CS35 (a positive control known to recognize AM and LAM from various mycobacterial strains [15]), or the murine IgG_2a_ mAb 9d8 (known to recognize only *M. tuberculosis* AM) [13, 15, 19] and processed essentially as reported elsewhere [24], with details described in the Supplementary Methods. We note that we used IgG_2a_ 9d8, a switch variant of the protective IgG_3_ mAb 9d8, which binds to *M. tuberculosis* AM in a similar manner as the parent mAb [19], for technical reasons, because it does not clump like the IgG_3_ isotype and is easier to purify.

### Phagocytosis Assay

BCG bacteria were grown in Middlebrook 7H9 and conjugated with fluorescein isothiocyanate (FITC). Human monocytic cells (THP-1) were differentiated into adherent macrophages and then incubated for 2 hours with heat-inactivated sera (10% in Roswell Park Memorial Institute 1640 medium). The FITC-labeled BCG was then added at a multiplicity of infection (MOI) of 20. Phagocytosis was evaluated by flow cytometry, and fluorescence from the noninternalized FITC-labeled BCG was quenched by treating the cells with trypan blue [25]. We coincubated macrophages and sera prior to BCG infection to allow simultaneous processing of many samples, which we considered important for data comparison. To ensure that our method was comparable to the standard approach, we performed experiments with BCG preopsonized with sera from 3 subjects. To study any potential additive effects of complement on phagocytosis, sera from 2 subjects were either not heat inactivated or were supplemented with 1% non–heat-inactivated serum from a healthy volunteer. We further obtained primary macrophages from PBMCs of 2 BCG-vaccinated and *M. tuberculosis*–uninfected donors as described previously [26], coincubated those with paired sera from 2 representative subjects, and infected them with FITC-BCG at MOI of 10. Further details are described in the Supplementary Methods.

### Mycobacterial Growth Assay

THP-1 cells coincubated with paired sera and infected with BCG at MOI of 10 were harvested, lysed, and plated on days 1 and 4. Colony-forming units were counted after incubation at 37°C for 3 and 4 weeks. Further details are described in the Supplementary Methods. Ab responses were further correlated to PBMC-based MGIAs performed at Oxford University because whole-blood assays, owing to variability in mycobacterial stocks and week-to-week performances of the assays, showed considerably more variation than PBMC-based assays, which were run in only 2 batches [18].

### Transmission Electron Microscopy (TEM) and Immunofluorescence

BCG (Pasteur) or *M. tuberculosis* (H37Rv) was grown in the presence and absence of 0.05% tyloxapol. For TEM studies, BCG strains were fixed as described previously [7]. For immunofluorescence studies, fixed BCG or *M. tuberculosis* was incubated with the murine mAbs IgG_1_ 24C5 to glucan [27], IgG_3_ CS35 to AM/LAM [15], isotype-matched controls, or human sera (1:100). Secondary Abs and further details are described in the Supplementary Methods.

### Assessment of Phagosome-Lysosome (P-L) Fusion

THP-1 macrophages were coincubated with 10% heat-inactivated paired sera from 2 subjects (chosen on the basis of high intracellular growth inhibition rates with postvaccination sera) and infected with Alexa 488–conjugated BCG [28] at a MOI of 20 for 1 hour, followed by incubation with 100 nM LysoTracker Red DND-99 (Life Technologies) for 1 hour as described elsewhere [29]. After washing and fixing, the cells were examined with a Leica SP5 confocal microscope, 14–18 images were taken for each sample, and a minimum of 50 BCG phagosomes were counted to quantify the percentage of phagosomes co-localizing with the LysoTracker. Further details are described in the Supplementary Methods.

### Statistical Analysis

Statistical analysis was performed using Prism software, version 6.04 (GraphPad). Comparisons were made using the nonparametric Wilcoxon matched-pairs signed rank test or the Mann–Whitney *U* test. Correlations were assessed by Spearman rank correlation. Correlations involving Ab responses to the 12 AM epitopes were adjusted for multiple comparisons, using the Holm-Bonferroni method [30].

## RESULTS

### Ab Responses Before and After BCG Receipt

**Figure 1.**
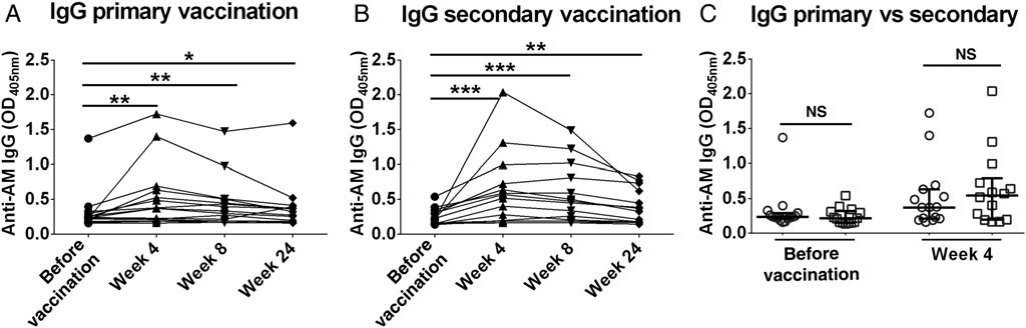
Immunoglobulin G (IgG) antibody (Ab) responses to arabinomannan (AM) before and after bacillus Calmette-Guerin (BCG) vaccination. *A* and *B*, IgG responses after primary vaccination (*A*) and after secondary BCG vaccination (*B*). The Wilcoxon matched-pairs signed rank test was used for analysis. *C*, Comparison of IgG responses between primary and secondary vaccination group, using the Mann–Whitney *U* test. Circles represent the primary vaccination group, and the squares represent the secondary vaccination group. Lines and error bars represent medians with interquartile ranges. *P < .05, **P < .01, ***P < .001, and ****P < .0001. Abbreviation: NS, not significant (*P* ≥ .05).

In both vaccination groups, compared with sera obtained before vaccination, sera obtained 4 and 8 weeks after vaccination had significantly higher IgG titers to AM (*P* < .01), which declined at 24 weeks (Figure 1*A* and 1*B*). Similar increases were observed for IgG_2_ responses (Supplementary Figure 1*A* and 1*B*), with a weak correlation between total IgG and IgG_2_ responses (r = 0.36, *P* = .05). While no significant differences were observed between the primary and secondary vaccination groups' total IgG responses before and 4 weeks after vaccination (Figure 1*C*), the secondary BCG vaccination group had significantly higher IgG_2_ titers at both time points (Supplementary Figure 1*C*), although the overall magnitude of IgG_2_ increase was similar between the groups. Increases in IgA and IgM titers to AM were only significant after secondary vaccination (Supplementary Figures 2 and 3). Of note, 1 subject (in the secondary vaccination group) with a prevaccination IgG reactivity of 4.9 SDs greater than the average was excluded as an outlier, while another subject (primary vaccination group) with a prevaccination value of 1.8 SDs above average was included, because we used an inclusion threshold of 2 SDs from the mean prevaccination value.

Because knowledge about the similarity in AM and LAM epitope recognition might have important implications, we tested IgG reactivities to AM isolated from the capsule of both BCG and *M. tuberculosis* H37Rv and correlated those to LAM isolated from the cell wall of *M. tuberculosis* H37Rv in postvaccination sera from 18 randomly chosen subjects (Supplementary Figure 4). The responses to LAM and AM correlated strongly (*P* < .0001), with correlations between LAM and AM isolated from the same strain (H37Rv) slightly stronger (r = 0.95) than those between AM and AM or LAM isolated from different strains (r = 0.88).

### Ab Responses to AM OS Epitopes

**Figure 2.**
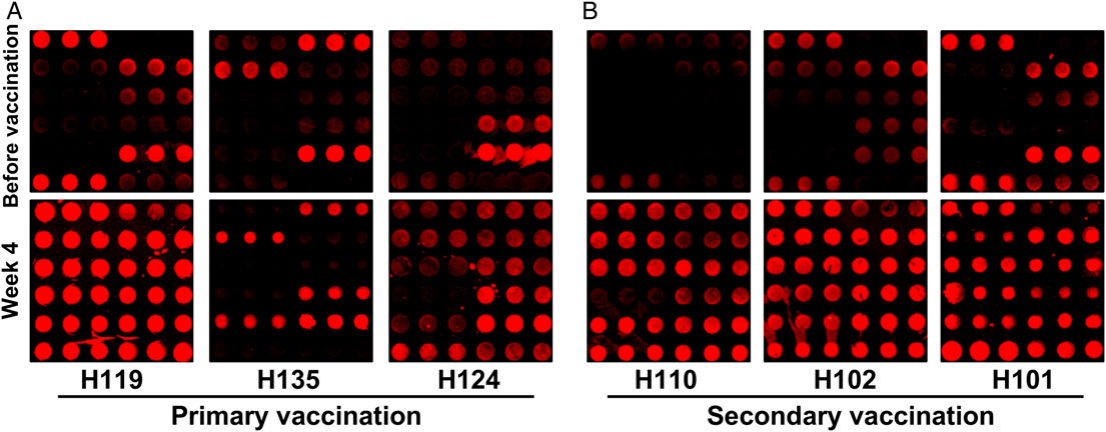
Immunoglobulin G (IgG) reactivity with a 12-member arabinomannan (AM) oligosaccharide (OS) microarray. Representative microarray images of sera obtained from 3 subjects in the primary (*A*) and secondary (*B*) vaccination groups before and 4 weeks after bacillus Calmette-Guerin (BCG) vaccination, demonstrating the heterogeneous patterns of reactivity to AM OS epitopes (printed in triplicates). For each subject, the brightness and contrast of the images were adjusted with the same settings, using GenePixPro6.1.

Regardless of vaccination group, subjects' sera reacted with AM epitopes in heterogeneous patterns (Figure 2). Overall, secondary vaccination appeared to induce more-pronounced IgG responses to AM OS epitopes than did primary vaccination (Supplementary Figure 5).

### Enhanced BCG Macrophage Phagocytosis With Postvaccination Sera

**Figure 3.**
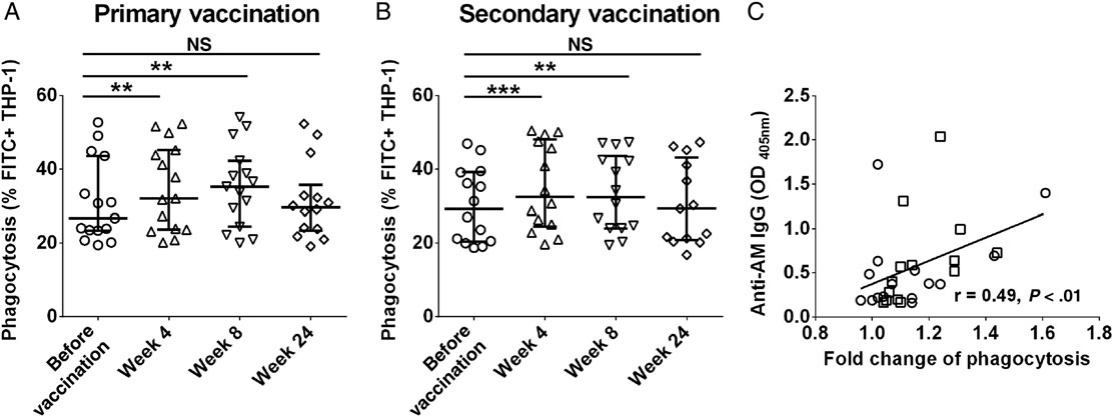
Effects of postvaccination sera on bacillus Calmette-Guerin (BCG) phagocytosis by human macrophages. Phagocytosis rates increased significantly upon coincubation of THP-1 cells with sera obtained 4 and 8 weeks after vaccination, compared with sera obtained before vaccination, but decreased to prevaccination levels upon coincubation with sera obtained 24 weeks after vaccination in both the primary (*A*) and secondary (*B*) vaccination groups. Lines and error bars represent medians with interquartile ranges. The Wilcoxon matched-pairs signed rank test was used for analysis. *P < .05, **P < .01, ***P < .001, and ****P < .0001. Of note, 2 subjects, one from the primary vaccination group and one from the secondary vaccination group, had insufficient serum volume 24 weeks after vaccination and thus have no phagocytosis data for this time point. *C*, Significant correlation between immunoglobulin G (IgG) responses to arabinomannan (AM) 4 weeks after vaccination and enhanced BCG phagocytosis (in fold change compared to coincubation with prevaccination sera) by THP-1 cells coincubated with corresponding sera obtained 4 weeks after vaccination, using Spearman rank correlation test. Circles represent the primary vaccination group, and squares represent the secondary vaccination group. Abbreviations: FITC, fluorescein isothiocyanate; NS, not significant (*P* ≥ .05).

For both vaccination groups, the THP-1 BCG phagocytosis increased significantly upon coincubation with sera obtained 4 and 8 weeks after vaccination (*P* < .01) but declined with sera obtained 24 weeks after vaccination (Figure 3*A* and 3*B*). Similar proportional enhancements of BCG phagocytosis were observed when comparing the standard method of preopsonizing BCG to our method of coincubating macrophages with sera before BCG infection (Supplementary Figure 6) and when using PBMC-derived macrophages (Supplementary Figure 7). However, with the addition of complement we found no considerable differences in phagocytosis between prevaccination and postvaccination sera (Supplementary Figure 8).

### Correlations Between BCG Phagocytosis and IgG Responses to AM and AM OS Epitopes

**Figure 4.**
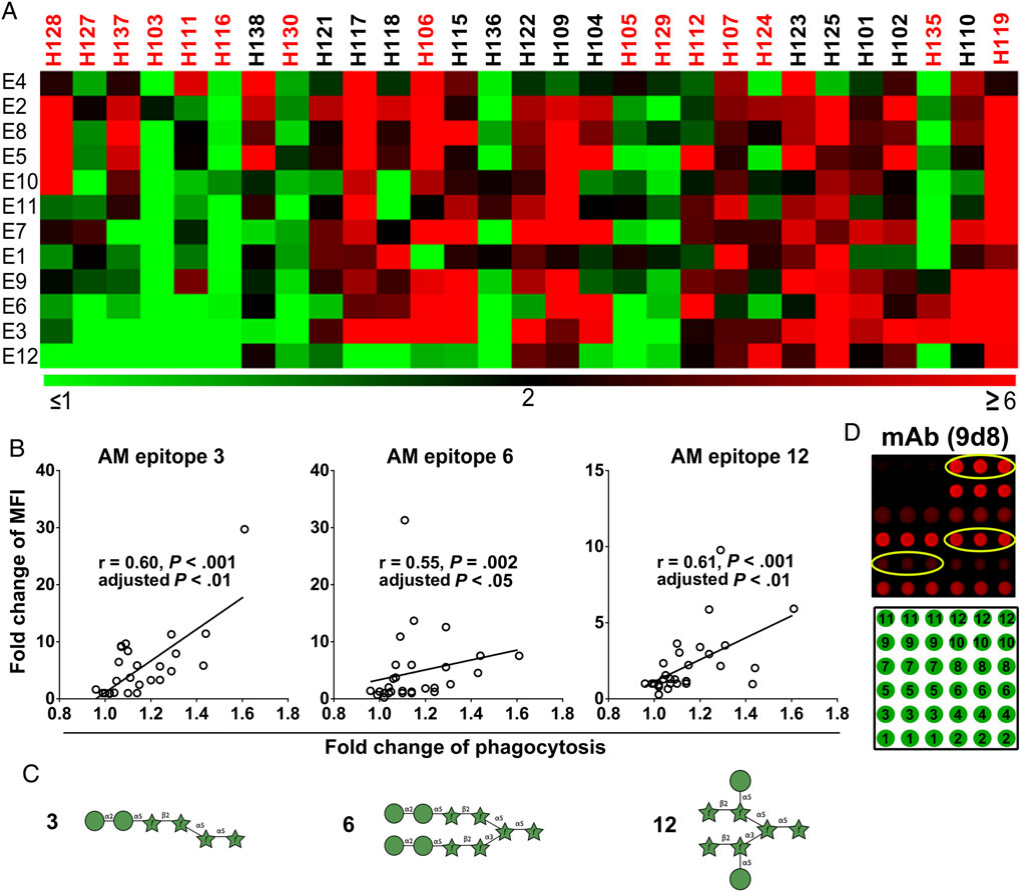
Enhancement of bacillus Calmette-Guerin (BCG) phagocytosis correlated with arabinomannan (AM)–epitope specific immunoglobulin G (IgG) responses. *A*, Heat map showing enhanced phagocytosis associated with antibody (Ab) responses to certain AM epitopes. The heat map was generated using the software MultiExperiment Viewer (available at: http://www.tm4.org/mev.html). The color feature represents fold change of median fluorescence intensity (MFI; from ≤1 to ≥6) with sera obtained 4 weeks after vaccination, compared with prevaccination sera. The subjects lined from left to right showed enhancement of BCG phagocytosis by THP-1 cells when coincubated with sera obtained 4 weeks after vaccination, compared with prevaccination sera (from low to high). Primary vaccination subjects' identifiers are listed in red, and those for subjects in the secondary vaccination group are in black fonts. *B*, Significant correlations between increased IgG reactivity to certain AM epitopes 4 weeks after vaccination and enhanced BCG phagocytosis by human macrophages coincubated with corresponding sera. Analysis was performed by the Spearman rank correlation test and the Holm-Bonferroni correction for multiple comparisons. *C*, Structures of AM epitopes 3, 6, and 12. Of note, all 3 segments contained at least 2 residues of mannose (green circles). Symbols used are those developed by the Consortium for Functional Glycomics, with green circles denoting D-mannose and green stars denoting D-arabinose. *D*, AM oligosaccharide fragments (printed in triplicates) recognized by switch-variant IgG_2a_ of the protective IgG_3_ monoclonal Ab (mAb) 9d8 against *Mycobacterium tuberculosis* AM. Positions of fragments on the microarray are shown below.

Increases in IgG reactivity to native AM by sera obtained 4 weeks after vaccination correlated significantly with increases in BCG phagocytosis when incubating THP-1 cells with the corresponding sera (*P* < .01; Figure 3*C*). The enhanced phagocytosis was strongly associated with increased IgG reactivity to certain AM OS epitopes, namely fragments 3, 6, and 12 (*P* < .05; Figure 4*A* and 4*B*). Interestingly, all 3 AM OS contained at least 2 residues of mannose (Figure 4*C*), and 2 of 3 epitopes (fragments 6 and 12) were also recognized by the murine IgG2a mAb 9d8 to *M. tuberculosis* AM (Figure 4*D*) [13, 19]. Of note, several other segments not associated with phagocytosis also contained mannose (Supplementary Figure 9).

### Effects of Postvaccination Sera on Intracellular Mycobacterial Growth Reduction

**Figure 5.**
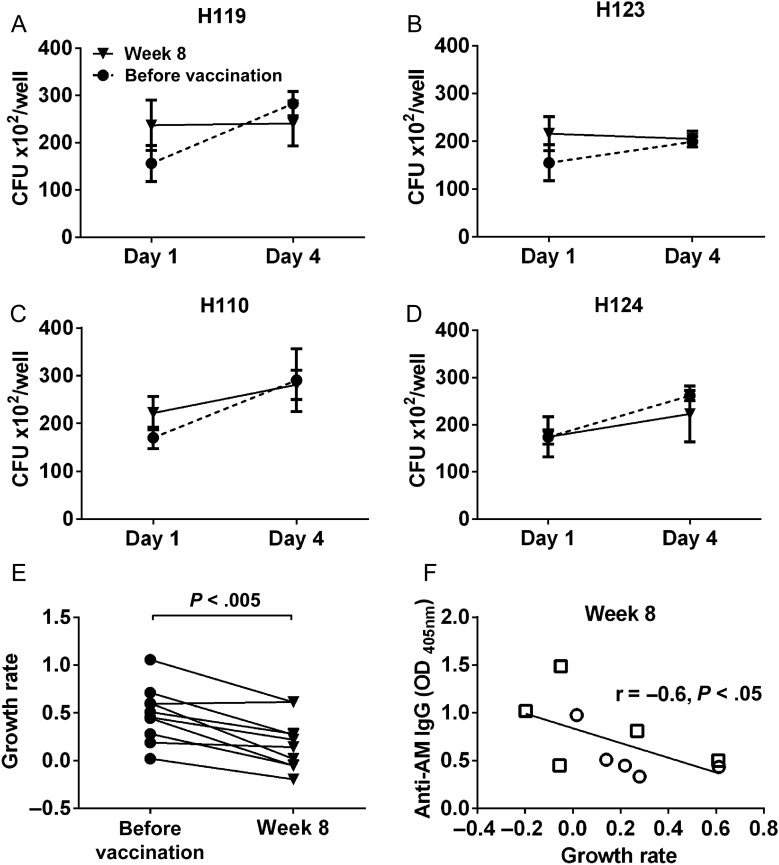
Bacillus Calmette-Guerin (BCG) growth in human macrophages incubated with postvaccination sera compared to prevaccination sera. THP-1 cells incubated with heat-inactivated sera obtained before and 8 weeks after vaccination from 10 subjects were infected with BCG (multiplicity of infection, 10) on day 1 and harvested for colony-forming unit (CFU) counting on days 1 (3 hours after infection) and 4. Of note, sera obtained 8 weeks after vaccination were used because insufficient quantity was left of sera obtained 4 weeks after vaccination. The 10 subjects were selected on the basis of the highest increase of BCG phagocytosis with THP-1 cells incubated with postvaccination sera relative to those incubated with prevaccination sera. *A–D*, Representative BCG growth in THP-1 cells incubated with paired sera (subjects H119 and H124 are from the primary vaccination group, and subjects H110 and H123 are from the secondary vaccination group). *E*, Significant BCG growth reduction in THP-1 cells incubated with postvaccination sera as compared to prevaccination sera. Because the initial amount of internalized BCG was different between most paired prevaccination and postvaccination sera, the growth rate was assessed by the proportion of increased bacteria relative to day 1 and was calculated as [CFU at day 4 – CFU at day 1]/CFU at day 1. Comparisons were made using the Wilcoxon matched-pairs signed rank test. The *n* value was limited to the top 10 phagocytosis-enhancing sera to allow for the processing of all sera in 1 experiment, which was performed at 2 separate time points. *F*, Correlation between immunoglobulin G (IgG) titers 8 weeks after vaccination and mycobacterial growth reduction, using the Spearman rank correlation test. Circles represent the primary vaccination group, and squares represent the secondary vaccination group. Abbreviation: AM, arabinomannan.

Significantly lower BCG growth rates were seen in THP-1 cells treated with postvaccination sera as compared to prevaccination sera (*P* < .01; Figure 5*A*–*E*). Moreover, BCG growth reduction correlated significantly with postvaccination IgG titers to AM (*P* < .05; Figure 5*F*). Because the number of paired sera tested was limited to 10 for practical reasons, correlations of IgG reactivities to AM OS epitopes lacked statistical power and were thus nonsignificant.

Ab responses were further correlated to PBMC-based MGIAs, for which data were available for 17 subjects, 8 from the primary and 9 from the secondary vaccination group [18]. At 4 weeks after vaccination, mycobacterial growth inhibition showed a significant correlation with IgG responses to AM (*P* < .01; Supplementary Figure 10).

### TEM and Immunofluorescence Studies

**Figure 6.**
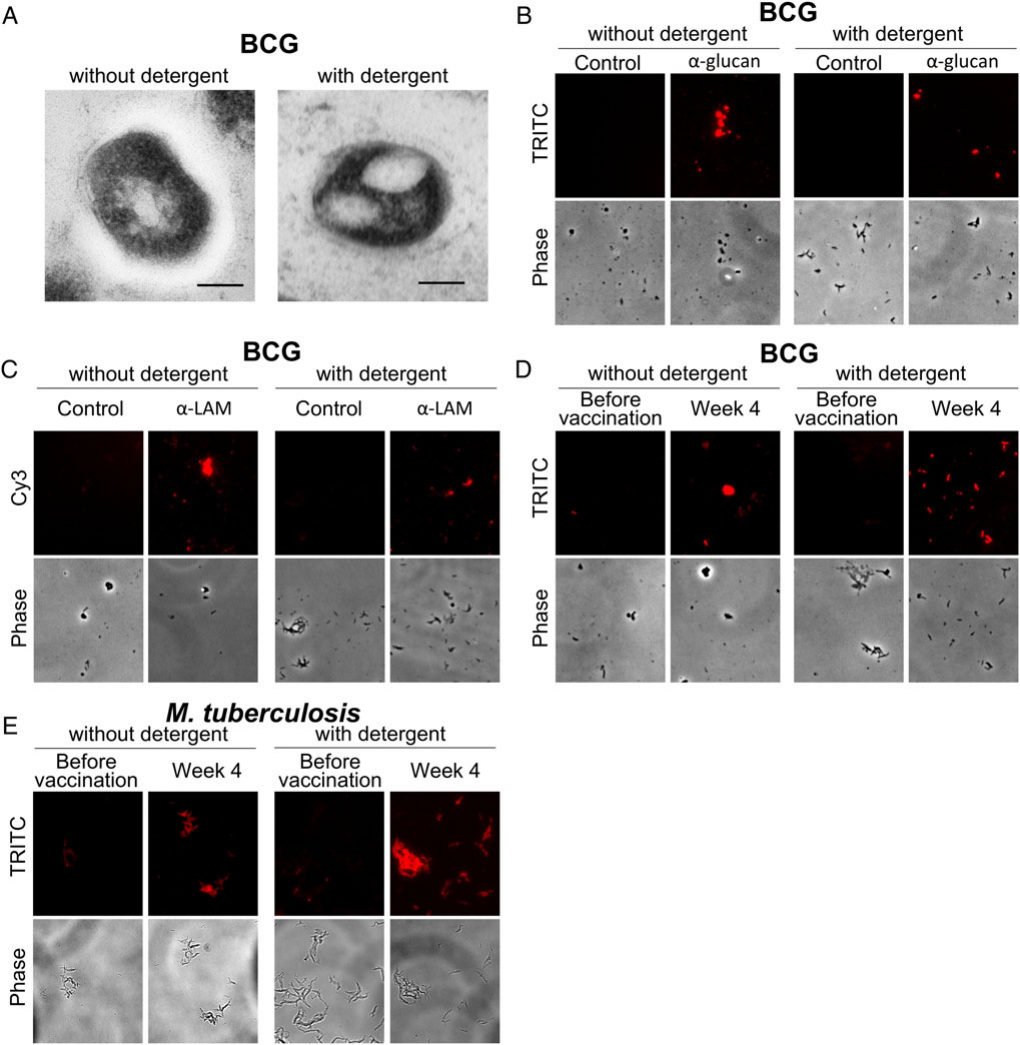
Transmission electron microscopy and immunofluorescence studies of mycobacteria grown without and with detergent. *A*, A visible electron-transparent zone is surrounding bacillus Calmette-Guerin (BCG) grown without detergent but not with detergent, indicating reduced capsule formation in the presence of detergent. The scale bar denotes 100 nm. *B*–*D*, Immunofluorescence microscopy of BCG incubated with control monoclonal antibody (mAb) 2D10 (immunoglobulin G_1_ [IgG_1_] against the *Cryptococcus neoformans* capsular polysaccharide glucuronoxylomannan) and anti-glucan IgG_1_ mAb 24C5 (*B*), with control mAb (IgG_3_, clone B10 of unknown specificity) and anti-LAM IgG_3_ mAb CS35 (*C*), and with prevaccination and postvaccination sera from subject H119 (*D*). *E*, Immunofluorescence microscopy of *Mycobacterium tuberculosis* (H37Rv) incubated with prevaccination and postvaccination sera from subject H119. Paired sera from this subject were selected on the basis of a high postvaccination IgG titer to arabinomannan, combined with enhanced postvaccination phagocytosis. In each panel of *A*–*D*, and in each sub panel of *E*, the brightness and contrast of the images were adjusted with the same settings, using ImageJ 1.48. Abbreviation: *M. tuberculosis, Mycobacterium tuberculosis.*

Consistent with other studies, the capsule was partially stripped off the bacteria when detergent was added to the culture medium [27, 31], and a thinner but still visible zone was observed surrounding the detergent treated bacteria (Figure 6*A*). Immunofluorescence microscopy showed that mAbs to α-glucan and AM/LAM bound to both BCG grown with and BCG grown without detergent, while an irrelevant isotype-matched control mAb did not (Figure 6*B* and 6*C*). Importantly, opsonization of BCG and *M. tuberculosis* grown under both culture conditions was considerably stronger with postvaccination serum as compared to prevaccination serum (Figure 6 *D* and 6*E*).

### P-L Fusion

**Figure 7.**
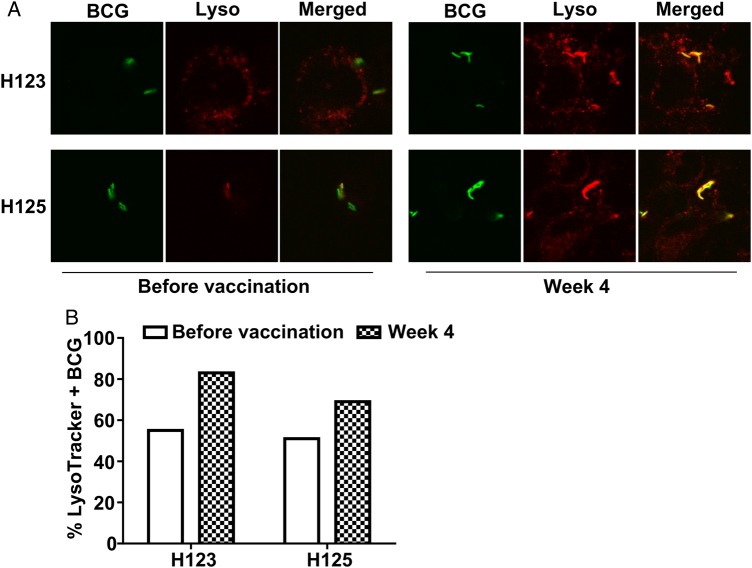
Effect of postvaccination sera on phagosome-lysosome (P-L) fusion in bacillus Calmette-Guerin (BCG)–infected THP-1 cells. *A*, THP-1 cells coincubated with sera obtained from 2 subjects (H123 and H125) before and 4 weeks after vaccination, followed by infection with Alexa 488–conjugated BCG at a multiplicity of infection of 20, were labeled with LysoTracker probes and analyzed by confocal microscopy. Images show BCG (green) localized within phagosomes, lysosomes (red), and colocalization of BCG/phagosomes and lysosomes (yellow) indicating P-L fusion. *B*, The percentage of LysoTracker + BCG/phagosomes (yellow) was considerably higher with postvaccination sera, relative to that with prevaccination sera, suggesting that the increased antibody reactivity to arabinomannan could reduce intracellular mycobacterial growth via FcɣR-mediated increase in P-L fusion.

To determine Ab effects on P-L fusion, we assessed paired sera from 2 representative subjects and found that postvaccination sera as compared to prevaccination sera enhanced P-L fusion (Figure 7).

## DISCUSSION

Our studies show that primary and secondary BCG vaccination in humans elicit serum IgG responses to the mycobacterial capsular polysaccharide AM that correlate strongly and significantly with responses to the cell wall glycolipid LAM. In accordance with prior studies investigating Ab reactivities to LAM [16], we demonstrate enhanced BCG opsonophagocytosis with postvaccination sera and show that the increased IgG reactivities to AM are significantly associated with enhanced BCG phagocytosis and growth reduction by human macrophages when incubated with the corresponding sera. Our data suggest that these associations might be driven by IgG responses targeting 3 specific AM epitopes. Interestingly, 2 of these epitopes are also strongly recognized by the murine mAb 9d8, which targets *M. tuberculosis* AM. These results are important in view of our previous results demonstrating that mAb 9d8 (IgG_3_) was protective against *M. tuberculosis* infection in mice [13], and contribute further supportive evidence for a role of Ab-mediated immunity in tuberculosis.

AM and LAM are major components of the mycobacterial cell envelope, and both have critical roles in tuberculosis pathogenesis [32]. Using a variety of AM-binding murine mAbs, data from our prior studies suggest that human and murine Abs against AM are diverse and heterogeneous [33]. We now provide additional data, showing that sera from *M. tuberculosis* uninfected adults recognize AM OS epitopes in heterogeneous patterns before and after primary or secondary BCG vaccination. These results suggest that individual prior exposure to environmental mycobacteria and BCG vaccination, and possibly also genetic influences on immune responses [34], could influence the repertoire of AM epitopes recognized. They further indicate the importance of using well-defined, homogenous glycans in preference to heterogeneous polysaccharides isolated from nature in observational, mechanistic, and vaccination studies.

The low IgG levels to AM prior to BCG vaccination in both primary and secondary vaccination groups, as well as their decline to near prevaccination levels at 24 weeks in both vaccination groups, are consistent with the often less robust and short-lived Ab responses to carbohydrate antigens and the need for conjugate vaccines to elicit lasting Abs [35]. Subjects in the revaccination group had significantly higher IgG_2_ titers before and after secondary BCG vaccination than subjects in the primary vaccination group but overall similar magnitudes in IgG_2_ responses. These results indicate that prior BCG vaccination could lead to ultimately higher titers of IgG_2_ to AM. The relevance of this finding remains to be explored because there is little evidence that BCG revaccination confers additional protection against tuberculosis [36, 37]. The weak correlation between total IgG and IgG_2_ responses to AM suggests that other IgG subclass responses against AM are also elicited, consistent with our prior human data [10], and the observation that IgG subclass responses to carbohydrates are not restricted to IgG_2_ [38]. Although not the focus of this study, we note that the induction of mucosal in addition to systemic AM-specific Abs could have important implications for vaccine efficacy. It is, consequently, noteworthy that oral BCG vaccination was shown to induce LAM-reactive secretory IgA [39]. We found a broad range of increases in AM-specific Ab titers after BCG vaccination, which, along with the epitope-specific heterogeneity, is in line with the frequently observed individual variation of Ab responses to mycobacterial antigens [40]. Whether the heterogeneity in Ab titers and epitope-specific responses, and/or the rapid decline of vaccine induced Abs, could be associated with the limitations of BCG vaccine effectiveness warrants further exploration.

The significant correlation between the postvaccination AM-specific IgG titer and the enhanced BCG phagocytosis and mycobacterial growth reduction in macrophages when coincubated with the corresponding sera suggest that these associations are FcγR mediated. These results are consistent with data from de Valliere et al, who demonstrated that Abs to LAM induced by BCG vaccination were associated with enhanced mycobacterial internalization and growth inhibition by primary human macrophages and neutrophils [16]. The increase in P-L fusion in macrophages infected with BCG preopsonized with postvaccination sera supports that our observed enhanced effects are FcɣR mediated. These data are in accordance with other studies, such as that by Armstrong et al, who demonstrated that rabbit tuberculosis-immune sera opsonize *M. tuberculosis* and promote macrophage phagocytosis, as well as P-L fusion [41]; that by Kang et al, who showed that entry of ManLAM beads into macrophages via mannose receptors inhibited P-L fusion while entry of anti-LAM mAb coated beads via FcɣRs did not [42]; and that by Kumar et al, who recently demonstrated that human natural opsonizing Abs promote phagosome maturation and restrict *M. tuberculosis* survival in human macrophages [43].

We anticipated that some of our observed Ab-associated effects would have been further increased in the presence of complement because human IgG can enhance complement activation and increase phagocytosis of BCG and *M. tuberculosis* by macrophages [44, 45]. Interestingly, in the presence of complement, phagocytosis was similarly and considerably enhanced with both prevaccination and postvaccination sera. Prior studies have shown enhanced phagocytosis via complement receptors with nonimmune sera [44, 46], but to our knowledge this has not been associated with reduced intracellular growth or enhanced P-L fusion. Further studies to elucidate the effects of combined activation of complement and FcɣRs on intracellular *M. tuberculosis* survival are needed but were beyond the scope of this study. We also made the interesting observation that PBMC MGIA results 4 weeks after vaccination [18] correlated significantly with the IgG titers to AM in the corresponding sera. A possible explanation could be that the PBMCs may have been coated with AM-specific Abs, leading to better uptake and killing of mycobacteria by phagocytic cells, although the portion of such Abs would almost certainly be small. Another possibility is that BCG induces both cell-mediated and humoral immunity.

Glycan microarrays, a recently developed technology [24, 47], have enabled the identification of Abs that recognize immunologically relevant OS fragments from organisms such as *Salmonella* and malaria parasites [47, 48]. Here, the use of novel mycobacterial glycan microarrays has enabled us to delineate the spectrum of Ab responses elicited to AM OS fragments. Our data suggest that Abs targeting specific AM OS epitopes could be more protective than Abs targeting other AM epitopes—a hypothesis supported by the observation that 2 of 3 epitopes recognized by human sera overlap with those recognized by the switch variant of the murine protective mAb 9d8 [13, 19]. Further characterization of human Abs recognizing specific AM OSs and the generation of mAbs against these epitopes are now warranted, to elucidate the specific mechanisms of such Abs in vitro and in vivo.

In accordance with other experimental studies, we cultured BCG in medium supplemented with detergent to avoid heavy bacterial clumping—an approach required for the feasibility of our experiments, with the downside of removing the mycobacterial capsule to a considerable extent [27, 31]. We attempted to investigate whether subjects' Abs targeted AM of any potentially remaining capsular fraction or the related motifs present in cell wall–associated LAM [32]. Consistent with other EM studies, we saw a clear reduction of the capsule from BCG grown with detergent as compared to without detergent [31]. Nevertheless, BCG grown under both conditions was reactive with a mAb directed against capsular α-glucan [9, 27], suggesting that some components of the capsule remained. This observation was consistent with our prior data showing that the anti-glucan mAb binds to *M. tuberculosis* strains grown under both conditions with only slightly greater apparent affinity for *M. tuberculosis* grown without detergent [27]. Thus, the subjects' Abs could have bound to capsular AM and/or the cell wall–associated AM portion of LAM, which is supported by the observation that IgG responses to capsular AM and cell wall LAM correlated strongly and significantly. Of note, when isolated from the same strain, correlations between reactivities to AM and LAM were even stronger than correlations between AM isolated from different strains, supporting that the surface expression of AM is strain dependent [19, 49, 50]. We further showed that postvaccination but not prevaccination sera were reactive with BCG and with *M. tuberculosis* grown in both the presence and absence of detergent, confirming that Abs elicited through BCG vaccination can opsonize both strains, regardless of culture conditions.

In summary, our studies show that BCG vaccination induces Abs to the mycobacterial capsular polysaccharide AM in humans and that these are strongly correlated with reactivities to the cell wall glycolipid LAM. In accordance with prior studies, we show that these Ab responses are associated with defense mechanisms against mycobacterial infection and provide further evidence indicating that they are FcɣR mediated. Importantly, this work is the first to demonstrate that such Ab responses display a heterogeneous recognition pattern of specific AM OS epitopes. Our results demonstrate that these Abs are biologically active in that they can promote mycobacterial opsonization, phagocytosis, and intracellular growth reduction, and enhance P-L fusion. Our data support and complement previous experimental and clinical studies demonstrating the relevance of Abs targeting AM, specifically some of its OS epitopes, and highlight the importance of further mechanistic studies with mAbs against specific AM glycan structures.

## Supplementary Data

Supplementary materials are available at http://jid.oxfordjournals.org. Consisting of data provided by the author to benefit the reader, the posted materials are not copyedited and are the sole responsibility of the author, so questions or comments should be addressed to the author.

Supplementary Data
