# Supplementary material for: Association of Human Antibodies to Arabinomannan With Enhanced Mycobacterial Opsonophagocytosis and Intracellular Growth Reduction
Source: J Infect Dis. 2016 Apr 7;214(2):300–10. doi: 10.1093/infdis/jiw141 (PMC4918826; doi:10.1093/infdis/jiw141)
Supplement: Supplementary Data [file supp_214_2_300__index.html]

Association of Human Antibodies to Arabinomannan With Enhanced Mycobacterial Opsonophagocytosis and Intracellular Growth Reduction — Association of Human Antibodies to Arabinomannan With Enhanced Mycobacterial Opsonophagocytosis and Intracellular Growth Reduction — Supplementary Data 

# Association of Human Antibodies to Arabinomannan With Enhanced Mycobacterial Opsonophagocytosis and Intracellular Growth Reduction

## Supplementary Data

Supplementary Data

- Supplementary Data - Docx file
- Supplementary Figures - docx file
