# Supplementary material for: Association of Human Antibodies to Arabinomannan With Enhanced Mycobacterial Opsonophagocytosis and Intracellular Growth Reduction
Source: J Infect Dis. 2016 Apr 7;214(2):300–10. doi: 10.1093/infdis/jiw141 (PMC4918826; doi:10.1093/infdis/jiw141)
Supplement: Supplementary Data [file supp_jiw141_jiw141supp.docx]

**Supplementary Methods**

**ELISAs.** ELISAs were performed as described [10]. Briefly, microtiter plates (Maxisorp) were coated with AM or LAM at 10 μg/ml. Serum samples (1:50) were added and bound Abs were detected with protein A-alkaline phosphatase (AP) for IgG (Sigma), mouse anti-human IgG_2_-AP for IgG_2_ (Southern Biotech), goat anti-human IgA-AP for IgA (Sigma), and mouse anti-human IgM-AP for IgM (Southern Biotech), followed by p-nitrophenyl phosphate substrate. Plates were washed with TBS containing 0.1% Tween 20 between all ELISA steps. The optical densities (OD) were measured at 405 nm.

**Ab detection by microarray.**

Microarray slides were blocked with 3% BSA in PBS at 4 °C overnight. The slides were then incubated with diluted sera (1:100), the murine mAb CS35 (5 μg/ml) or the murine IgG_2a_ mAb 9d8 at 37 °C for 4 hours (hrs). After washing with PBST, the slides were sequentially incubated with goat anti-human biotin-labeled IgG or goat anti-mouse biotin-labeled IgG (Southern Biotech, AL; Jackson Immunoresearch, PA) and incubated with a streptavidin probe tagged with SureLight®P3 Cy5 (Cayman Chemicals, MI) at 37 °C for 2 hrs. After the final wash, the slides were dried by spinning the slide for 5min at 200xg, and then scanned using the GenePix 4000 Microarray scanner system (Molecular Devices, CA). Images were analyzed by the image-processing software Spotfinder (http://www.tm4.org/spotfinder.html), which measured median pixel intensity (MPI) and neighboring background pixel intensity (BPI) of individual spots. The median fluorescent intensity (MFI), representing AM-epitope specific Ab responses, was the MPI minus the BPI. The minimum value of pixel intensity was determined by the MFI of the spots with low quality, which was determined by the software quality control score for each spot depending on signal-to-noise ratio and spot shape. The final MFI was averaged from the triplicates.

**Phagocytosis assay**

BCG was grown to OD_600_ of 0.5-1.0 in Middlebrook 7H9 broth supplemented with 0.05% tyloxapol and oleic albumin dextrose catalase enrichment. To conjugate with FITC, 1 ml of bacteria (5x10^9^) was incubated with 0.2 mg FITC in PBS (pH 7.4) at room temperature for 1 hr. The unbound FITC was removed by washing the bacteria three times with PBS. To remove bacterial clumps, the FITC-labeled bacteria were passed through a 25G-needle syringe 10 times as described [7], followed by centrifugation at 300 g for 5 mins. The supernatant consisting of mainly single-cell bacteria was used for infection. The final concentration of bacteria was assessed by measuring OD at 600 nm.

Human THP-1 monocytic leukemia cells (ATCC) were cultured in complete RPMI media (Life Technologies, CA), supplemented with GlutaMAX™ (Life Technologies, CA), penicillin, streptomycin and 10% fetal bovine serum (FBS; Atlanta Biologicals, GA). One day prior to infection, the cells were placed in 96-well tissue culture plates at a density of 1x10^5^ cells/well in complete RPMI without antibiotics. To differentiate the cells into adherent MΦ, media were supplemented with 50 nM phorbol 12-myristate 13-acetate (PMA, Sigma, MO). On the day of infection, the THP-1 monolayers were washed extensively to remove unattached cells. Cells were then incubated for 2 hrs at 37 °C in media supplemented with heat-inactivated sera from subjects’ pre- or post-vaccination time points (10%; 30 mins at 56 °C). The serum was added in duplicates to a 96-well plate. The FITC-conjugated BCG was added at a multiplicity of infection (MOI) of 20, and the monolayers were incubated with the bacteria for 3 hrs. Afterwards, the monolayers were washed with PBS, and dissociated into cell suspension by treatment with Cellstripper Dissociation Reagent (Corning Fisher). The phagocytosis was evaluated by flow cytometry, and fluorescence from the non-internalized membrane-bound FITC-labeled BCG was quenched by treating the cells with 0.2% trypan blue for 10 min. The percentage of cells exhibiting FITC fluorescence was representative of the percentage of cells with BCG phagocytosis.

To pre-opsonize bacteria, 6x10^6^ BCG diluted in 30 µl RPMI were mixed with 15 µl of undiluted subject's serum and incubated at 37 °C for 45 mins. The bacteria were spun down, washed with RPMI, and re-suspended in 150 µl RPMI with 10% FBS. Fifty µl of pre-opsonized BCG suspension were added to 1x10^5^ THP-1 cells (MOI 20) in duplicates of each well to a 96-well plate, and incubated for 3 hrs. The cells were then harvested and examined by flow cytometry as described for the phagocytosis assay.

**Human primary blood monocyte-derived macrophage infection.**

Peripheral blood mononuclear cells (PBMCs) from two healthy volunteers with a history of BCG infection and no evidence of latent Mtb infection (negative Tuberculin skin-test and/or interferon-gamma release assay) were purified from heparinized blood using Ficoll-Paque PLUS gradient centrifugation according to the manufacturer's instructions (GE Healthcare Bio-Sciences, PA) and basically as described (26). PBMCs were plated into 48-well plate at concentrations of 2x10^6^ cells/well, and 2 hrs later the non-adherent cells were removed by extensive washings with PBS. Adherent cells were incubated in RPMI supplemented with either 10% heat-inactivated autologous serum if Ab titers to AM were low or with type AB serum with low Ab titers against AM if Ab titer to AM in autologus serum was moderate-high, and 10 ng/ml M-CSF (R&D Systems, MN) for 7 days. On the day of infection, the MΦ monolayers were washed extensively. The cells in duplicates of each well of a 48-well plate were co-incubated with 10% HI sera diluted in RPMI media, and infected with FITC-conjugated BCG at MOI 10. After 2 hrs, the phagocytosis was evaluated by flow cytometry as described for THP-1 cells under phagocytosis assay***.***

**Human MΦ-based mycobacterial growth assay.** THP-1 cells were differentiated and infected essentially as described for the phagocytosis assay, except that BCG were added at an MOI of 10. At day 1, the cells were washed 3 hrs after infection, and treated with 100 µg/ml gentamycin for 1 hr to kill extracellular bacteria. For cells harvested on day 1, 0.05% SDS was added to lyse cells. The lysate was serially diluted and plated on Middlebrook 7H11 agar plates. For the cells harvested on day 4, 100 µl RPMI medium containing 10% human serum were added to each well on day 1, and the cells were lysed, pooled with supernatant, and plated on day 4. Colony forming units (CFU) were counted after incubation at 37 °C for 3 weeks, and counts were repeated at 4 weeks to check for additional visible colonies.

**TEM and IF**

To evaluate the impact of detergent on the formation of the mycobacterial capsule, BCG was grown in the presence and absence of 0.05% tyloxapol. For TEM studies, BCG cultures were mixed 1:1 with 4.0% paraformaldehyde and 5% glutaraldehyde, 0.1% ruthenium red in 0.2 M sodium cacodylate buffer, postfixed with 1% osmium tetroxide followed by 1% uranyl acetate, dehydrated through a graded series of ethanol, and embedded in LR white resin (Electron Microscopy Sciences, Hatfield, PA) [7]. Ultrathin sections were cut on a Reichert Ultracut UCT, stained with uranyl acetate followed by lead citrate, and viewed on a JEOL 1200EX transmission electron microscope at 80 kv.

For IF studies, BCG strains were fixed with 4% formaldehyde (PFA) for 15 mins at room temperature (RT), and H37Rv strains were fixed with 2% PFA at 4°C for 1hr. The different fixation method for Mtb was used according to our institutional regulations for experiments performed under BSL3 conditions. The fixed bacteria were washed with PBS, placed on poly-L-lysine slides (Sigma, MO) and air dried. Slides were blocked with 1% BSA/PBS (blocking buffer) for 1 hr at RT. Murine mAbs (10 µg/ml) IgG_1_ 24C5 against glucan [27], IgG_3_ CS35 against AM/LAM [15], isotype-matched controls (IgG_1_ 2D10 against the *Cryptococcus neoformans* capsular polysaccharide glucuronoxylomannan (GXM) [27]), IgG_3_ (clone B10 of unknown specificity (Southern Biotech, AL)), or human sera (1:100) were added, and slides incubated at 4 °C overnight. After washing, secondary Abs (10 µg/ml) tetramethylrhodamine (TRITC)-labeled goat anti-mouse IgG_1_ (Southern Biotech, AL), Cy3-labeled goat anti-mouse IgG (Jackson ImmunoResearch, PA), and TRITC-labeled goat-anti human IgG (Southern Biotech, AL) were added for 1.5 hrs at RT in the dark. After washing, the slides were mounted with 90% glycerol, and viewed with a Zeiss observer microscope.

**Assessment of phagosome-lysosome (P-L) fusion.**

THP-1 cells were plated into an 8-well Lab-Tek II chamber slide (Thermo Scientific, MA) at concentrations of 1x10^5^ cells/well, and differentiated into MΦ by PMA as described for phagocytosis assay. BCG was conjugated with Alexa 488 by incubating with 10 µg/ml Alexa Fluor® 488 NHS Ester (Life Technologies) at RT for 1 hr as described [28]. The Alexa 488-conjugated BCG was processed into single-bacteria suspension as described for FITC-conjugated BCG. The THP-1 cells incubated with RPMI supplemented with 10% HI paired sera from two subjects were infected by Alexa 488-conjugated BCG at MOI 20 for 1 hr, then washed with RPMI and incubated with RPMI supplemented with 10% FBS and 100 nM LysoTracker® Red DND-99 (Life Technologies) as described [29]. After 1 hr, the cells were washed with PBS, fixed with 4% PFA, and examined with a Leica SP5 confocal microscope. For each serum, 14-18 images were taken and a minimum of 50 BCG phagosomes were counted to quantify the percentage of BCG phagosomes that co-localized with the LysoTracker.
