# Supplementary material for: Association of Human Antibodies to Arabinomannan With Enhanced Mycobacterial Opsonophagocytosis and Intracellular Growth Reduction
Source: J Infect Dis. 2016 Apr 7;214(2):300–10. doi: 10.1093/infdis/jiw141 (PMC4918826; doi:10.1093/infdis/jiw141)
Supplement: Supplementary Data [file supp_jiw141_jiw141supp_figs.docx]

**

**

**Figure S1. Pre- and post-BCG vaccination IgG_2_ titers to AM. (A)** IgG_2_ responses after primary, and **(B)** secondary vaccination**.** Wilcoxon matched-pairs signed rank test. **(C)** Comparison of IgG_2_ responses between primary and secondary vaccination group using Mann-Whitney *U* test. Circles represent the primary, squares the secondary vaccination group. Lines and error bars represent medians with interquartile ranges. *: p < 0.05, **: p < 0.01, ***: p < 0.001. ****: p < 0.0001, ns: not significant (p ≥ 0.05).

**

**

**Figure S2. Pre- and post-BCG vaccination IgA titers to AM.** IgA responses after **(A)** primary and **(B)** secondary vaccination compared using Wilcoxon matched-pairs signed rank test. **(C)** Comparison of IgA responses between primary and secondary vaccination group using Mann-Whitney *U* test. Circles represent the primary, squares the secondary vaccination group. Lines and error bars represent medians with interquartile ranges. *: p < 0.05, **: p < 0.01, ***: p < 0.001. ****: p < 0.0001, ns: not significant (p ≥ 0.05).

**

**

**Figure. S3. Pre- and post-BCG vaccination IgM titers to AM.** IgM responses after **(A)** primary and **(B)** secondary vaccination compared using Wilcoxon matched-pairs signed rank test. **(C)** Comparison of IgM responses between primary and secondary vaccination group using Mann-Whitney *U* test. Circles represent the primary, squares the secondary vaccination group. Lines and error bars represent medians with interquartile ranges. *: p < 0.05, **: p < 0.01, ***: p < 0.001. ****: p < 0.0001.

**

**

**Figure S4. Correlations between post-vaccination IgG responses to AM and LAM.** Correlations between 8 weeks post-vaccination IgG responses in sera from 18 randomly chosen subjects to **(A)** *Mtb* (H37Rv) AM and BCG (Pasteur) AM; **(B)** *Mtb* **(**H37Rv) LAM and *Mtb* (H37Rv) AM; and (**C**) *Mtb* (H37Rv) LAM and BCG (Pasteur) AM using Spearman rank correlation test.

**

**

**Figure S5.** **Pre- and 4 weeks post-BCG vaccination IgG responses to AM epitopes using 12-member AM oligosaccharide microarray. (A)** Primary vaccination group, **(B)** Secondary vaccination group. *: p < 0.05, **: p < 0.01, ***: p < 0.001. ****: p < 0.0001, ns: not significant (p ≥ 0.05). The boxes show medians and interquartile ranges, the whiskers represent minimum to maximum values. Wilcoxon matched-pairs signed rank test.

**

**

**Figure S6. Effects on BCG phagocytosis when co-incubating macrophages with sera & BCG versus pre-opsonizing BCG with sera prior to macrophage infection.** Comparison between THP-1 cells co-incubated with 10% HI sera prior to infection with FITC-conjugated BCG and THP-1 cells infected with FITC-BCG pre-opsonized with HI sera. BCG phagocytosis by THP-1 cells was enhanced with post-vaccination sera regardless of method used and proportional increases observed were similar. The three subjects (H119 and H135 from the primary and H110 from the secondary vaccination group) were selected based on highest increase of BCG phagocytosis with THP1 cells with post- relative to pre-vaccination sera. The box and error bar represent mean and SD of phagocytosis rates from duplicates of two separate experiments.

**

**

**Figure S7. BCG phagocytosis by human primary monocyte-derived macrophages.** Human monocyte-derived macrophages obtained from two BCG vaccinated volunteers without latent *Mtb* infection were co-incubation with pre and post-vaccination sera from two subjects (H101 from the primary and H102 from the secondary vaccination group) followed by infection with FITC-conjugated BCG at MOI 10 for 2 hrs. Subjects were selected based on available remaining serum quantities and high increase of BCG phagocytosis in THP1 cells with post- relative to pre-vaccination sera. Similar to experiments with THP1 cells, the percentage of BCG phagocytosis observed with primary blood monocyte-derived macrophages was enhanced with co-incubation of 4 weeks post- compared to pre-vaccination sera. The box and error bar represent mean and SD of phagocytosis rates from duplicates.

**

**

**Figure S8. Effects of complement on BCG phagocytosis by macrophages.** THP-1 cells treated with 10% heat inactivated (HI), non-heat inactivated sera (N-HI), and HI sera supplemented with 1% N-HI serum from a non-BCG vaccinated, non-*Mtb* exposed volunteer with known low Ab reactivity to AM and other *Mtb* antigens, followed by infection with FITC-labeled BCG. Enhancement of phagocytosis with post-vaccination sera was only observed with HI sera. The box and error bar represent mean and SD of phagocytosis rates from duplicates of two separate experiments.

**
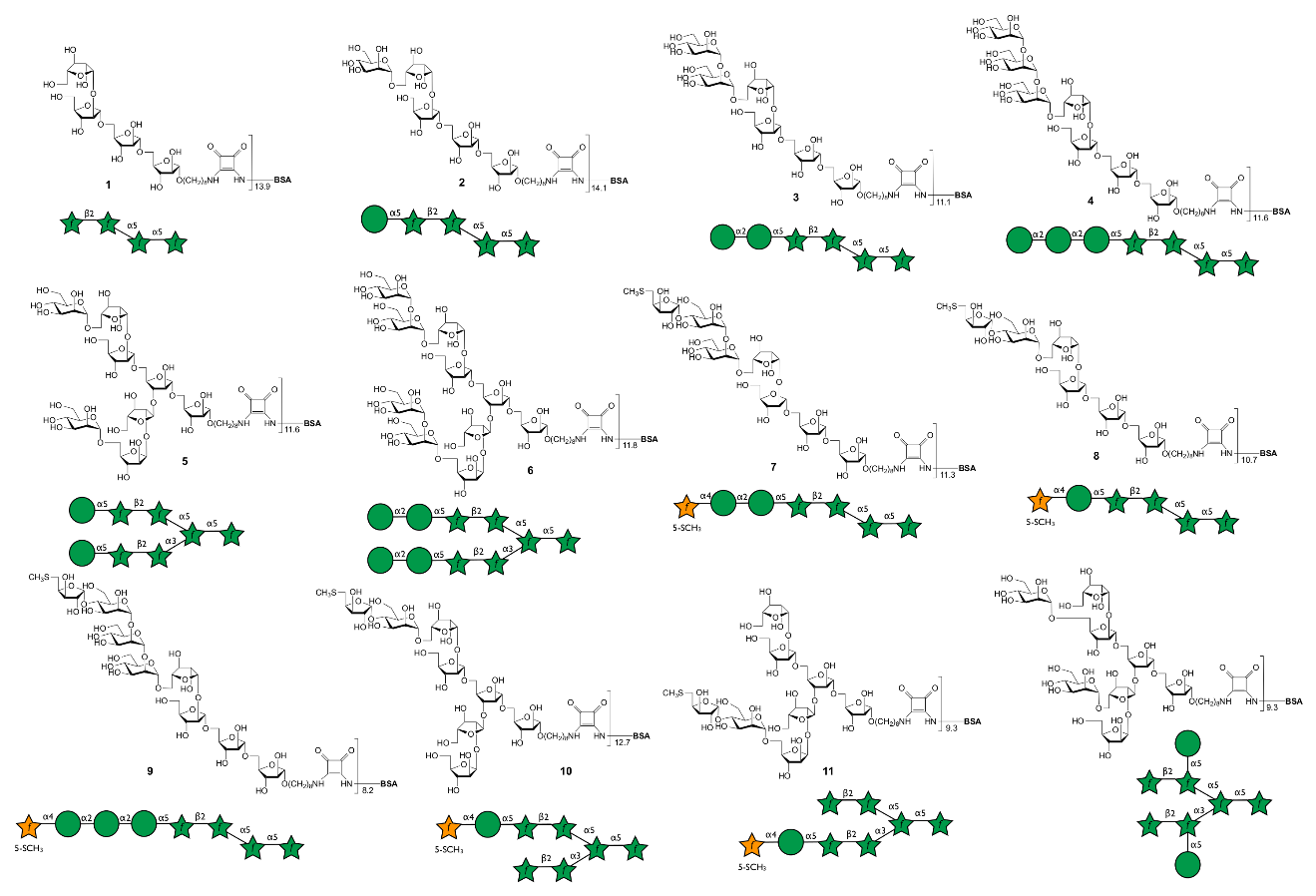
**

**Figure S9. Structures of 12 AM fragments included in glycan microarray.** Both line bond and pictorial structures are given. The loading of the oligosaccharides on the BSA is indicated adjacent to the bracket. Symbols used are those developed by the Consortium for Functional Glycomics [1]; green circles = D-mannose; green stars = D-arabinose; orange stars = D-xylose. The anomeric stereochemistry and linkage between residues is indicated by the Greek letter (α or β) and number between symbols. The ‘f’ inside some of the symbols indicates the furanose (five-membered ring) configuration for these residues; all other residues are in the pyranose (six-membered ring) configuration.

**
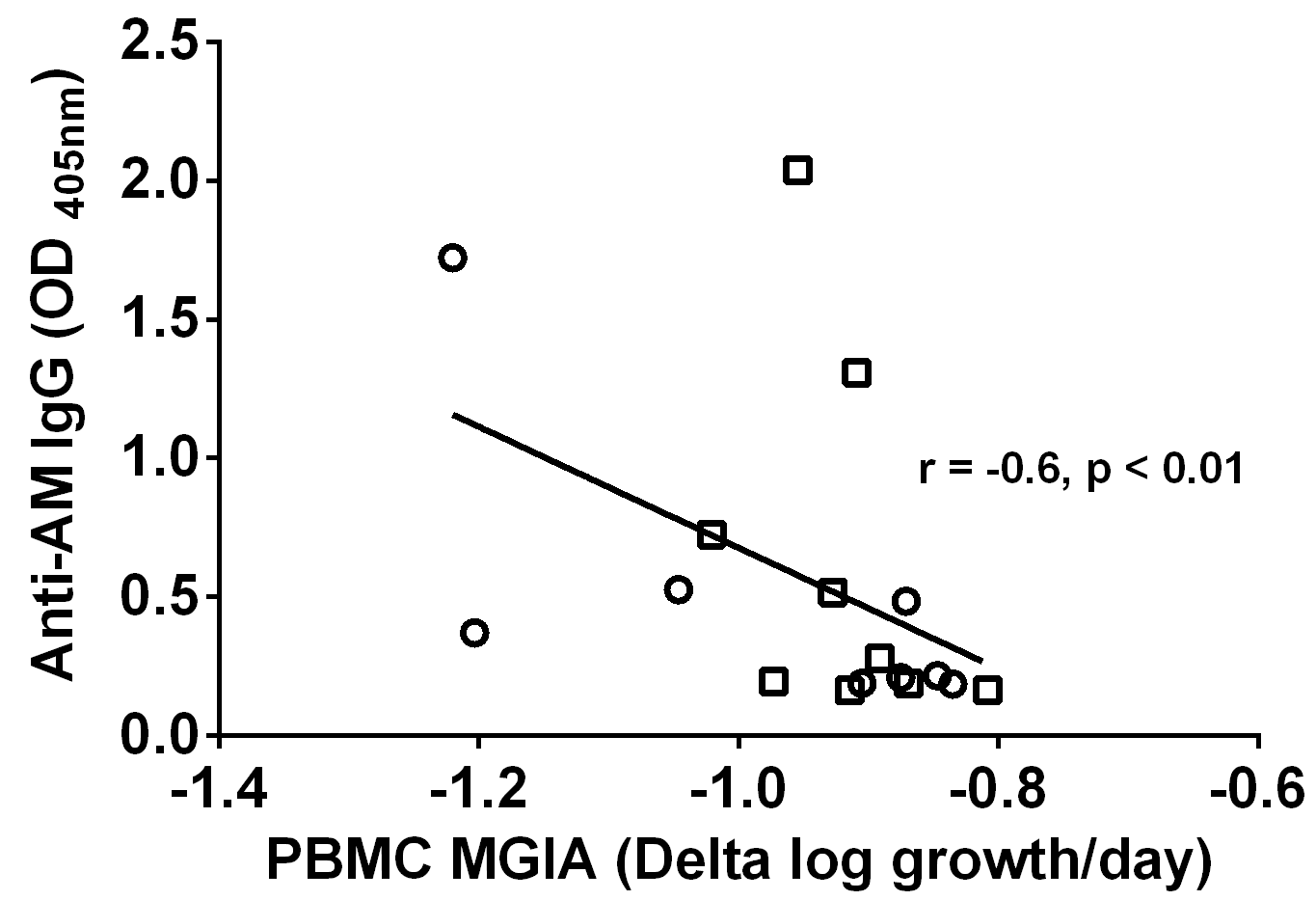
**

**Figure S10. Correlation between PBMC mycobacterial growth inhibition (MGIA) and IgG titers to AM at 4 weeks post vaccination.** PBMC MGIA data generated by Oxford University was available for 17 subjects from both the primary and the secondary vaccination group [2]. Circles represent primary vaccination group, squares the secondary vaccination group. Spearman rank correlation test.

**References**

1. Varki A, Cummings RD, Aebi M, et al. Symbol Nomenclature for Graphical Representations of Glycans. Glycobiology 2015; 25:1323-4.

2. Fletcher HA, Tanner R, Wallis RS, et al. Inhibition of mycobacterial growth in vitro following primary but not secondary vaccination with Mycobacterium bovis BCG. Clinical and vaccine immunology : CVI 2013; 20:1683-9.
